# Colorizing the Past: Deep Learning for the Automatic Colorization of Historical Aerial Images

**DOI:** 10.3390/jimaging8100269

**Published:** 2022-10-01

**Authors:** Elisa Mariarosaria Farella, Salim Malek, Fabio Remondino

**Affiliations:** 3D Optical Metrology (3DOM) Unit, Fondazione Bruno Kessler (FBK), Via Sommarive 18, 38123 Trento, Italy

**Keywords:** grayscale image colorization, historical photos, aerial images, deep learning

## Abstract

The colorization of grayscale images can, nowadays, take advantage of recent progress and the automation of deep-learning techniques. From the media industry to medical or geospatial applications, image colorization is an attractive and investigated image processing practice, and it is also helpful for revitalizing historical photographs. After exploring some of the existing fully automatic learning methods, the article presents a new neural network architecture, Hyper-U-NET, which combines a U-NET-like architecture and HyperConnections to handle the colorization of historical black and white aerial images. The training dataset (about 10,000 colored aerial image patches) and the realized neural network are available on our GitHub page to boost further research investigations in this field.

## 1. Introduction

Grayscale image colorization is an active research area stimulated by the latest achievements in artificial intelligence (AI) techniques and the exciting applications of colored data in many domains, from medicine to entertainment. Colorized images have been proven to support several image processing tasks (e.g., object recognition and classification) [1,2,3,4], besides helping with diagnostics [5,6,7], the movie industry [8], and many other fields.

Although manual image colorization has been explored since the 1980s, especially for old movies, fully automatic methods are relatively recent. The advent and the application, in particular, of deep-learning techniques to the colorization problem is facilitating this image processing activity. Currently, numerous deep-learning models have been proposed for converting grayscale images into color [9,10,11,12,13,14,15], mainly differing in the learning strategy and neural network architecture.

The advancement of these fully automatic methods is attractive for valorizing and enhancing historical photos, where colors can help (i) revitalize archival sources, (ii) improve the scene’s understanding, and (iii) support the analysis of past urban scenarios, landscapes, and settlements.

While terrestrial images capturing urban settings can be effective research and educational tools, historical aerial photos are incredible sources for investigating spatial changes. In the latter case, colorization was found to improve the images’ radiometric properties and to support further research activities, such as land cover mapping [16] or semantic segmentation [17]. Most of the existing learning-based models are mainly designed and trained to handle the colorization problem with terrestrial photos depicting (i) small urban or natural scenarios, (ii) human or animal subjects, or (iii) objects in outdoor or indoor contexts. Very few works have focused on developing appropriate learning-based models for colorizing historical aerial images [16,17,18] (Section 2.3), stored and preserved in large quantities in national and local archives and increasingly digitized worldwide. 

The current availability of many scanned aerial historical images is stimulating several research activities dedicated to fully exploring their capabilities for expanding geospatial knowledge, supporting multi-temporal analyses, and testing the effectiveness of modern automatic 2D and 3D processing algorithms. Available solutions for handling several digital image processing tasks are frequently ineffective with historical aerial data, primarily due to radiometric and quality issues. Among these unsolved tasks, the automatic colorization of grayscale aerial input data is still challenging and poorly investigated. 

The new learning-based architecture hereafter presented, Hyper-U-NET, contributes to bridging this research gap, besides supporting the community towards further analyses and implementations by sharing a consistent new training dataset for the colorization of aerial-scale imageries.

### Data and Paper Contribution

**Figure 1:**
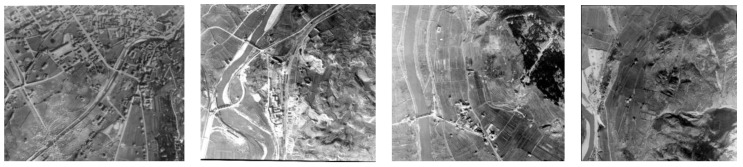
Examples of grayscale photos acquired from aerial platforms between 1944 and 1945 in Italy.

The article presents experiences and experiments on the automatic colorization of historical aerial images in order to increase their attractiveness and exploitation. The research activities are conducted within the TIME (hisTorical aerIal iMagEs) project (https://time.fbk.eu/ [accessed on 27 September 2022]) [19], supported by EuroSDR and several National Mapping Agencies (NMAs), to realize a benchmark of historical aerial images captured in European Countries since the 1950s (Figure 1). About 1000 grayscale images were collected and shared to stimulate geospatial investigations, and boost the testing and development of new automatic image processing algorithms.

While several algorithms are available for handling the colorization of grayscale images captured in urban contexts, investigations with aerial imageries are still limited. Therefore, our contribution and novelty focus on the following:

(a) Testing and evaluating the performance of several state-of-the-art and recent deep-learning models to colorize grayscale aerial images;
(b) Proposing a new methodology for colorizing historical aerial images based on a combination of a UNET-like network [20] and HyperConnections [21,22], including validation and ablation studies;
(c) Collecting and sharing a new benchmark dataset for colorizing historical aerial photographs (some 10,000 image patches).

## 2. Related Works

Despite a few differences, the existing colorization methods are mainly categorized in the literature [9,13] as user-guided (Section 2.1) and learning-based solutions (Section 2.2), differing in the level of operator intervention. 

Fully automatic deep-learning techniques have supplanted the more demanding traditional guided approaches, and are currently the most promising and explored methods for the image colorization task [13]. The following sections present an overview of both categories, reviewing more in-depth recent learning-based approaches and some implementations for the aerial-scale image case (Section 2.3). The benchmarking methods selected for tests and comparisons are summarized in Section 2.4.

### 2.1. User-Guided Approaches

Scribble and example-based methods are the most popular and investigated among the traditional user-guided approaches. 

In scribble-based solutions [23,24,25,26], some areas of grayscale images are annotated with scribbles of suited colors and then distributed until reaching the borders. In the pioneering work of Levin et al. [23], an optimization algorithm based on intensity similarities enabled better color propagation. Further optimizations of the scribble-based technique, in terms of time and chrominance distribution, have later been proposed [27,28,29,30]. While the adaptive edge extraction introduced by Huang et al. [27] allowed for reducing the colorization times and the color bleeding effects, a better chrominance assignment was achieved considering the intensity and texture similarities [29], or the color probability distribution [30]. The more recent user-guided approach proposed by Zhang et al. [2], instead, takes advantage of deep-learning architecture for minimizing the intervention time and improving the colorization results. The quality of the colorization results with these methods is related to the amount of scribbles provided. Color bleeding effects close to the edges are also frequent.

More limited user intervention is required in example-based approaches, where color pattern information is transferred from reference images to monochrome pictures [31,32,33,34,35]. In Welsh et al. [33], the luminance channel and neighborhood statistics are used to match the grayscale input, solving the color inconsistency and returning vivid color effects. This method has been further improved by Gupta et al. [36], exploring local features for improving pixel matching and transfer. The solution proposed by Li et al. [35] introduces a new location-aware cross-scale matching method, where error color matches are minimized. Nevertheless, the main limitation of all example-based approaches is the availability and quality of the reference images, often manually selected based on the subjects’ similarities. 

### 2.2. Deep Learning for Colorization

#### 2.2.1. Convolution Neural Networks (CNNs)

Convolutional neural networks (CNNs) are deep-learning algorithms consisting of multiple layers of small computational units working on small portions of the image. Different filters are applied to the previous layers in the convolution step, and some features are extracted from the input images, generating various “feature maps”. During the training process, CNNs learn the values of these filters. In recent years, these learning-based techniques have been widely adopted for solving the image colorization problem [22,37,38,39,40,41,42,43,44,45,46,47,48]. The implemented solutions mainly differ for the employed network architectures and loss functions.

Among the various CNN-based approaches, the methods proposed by Zhang et al. [42], Larsson et al. [22], and Iizuka et al. [43] are popular and are often reported in comparative studies.

The automatic colorization technique proposed by Iizuka et al. [43] combines both global priors and local image features. End-to-end learning leverages an existing large-scale classification dataset (Places) to learn the global priors discriminatively. The architecture can manage every resolution image (differently from many other algorithms). The method is intensely data-driven, and its effectiveness depends on the level of semantic similarity between images. In unclear situations, the model applies the dominant colors learned.

The fully automatic approach proposed by Zhang et al. [42] (unlike the user-guided solution presented in [2]) addresses colorization as a classification task. Class-rebalancing is applied during the training to increment color diversity, and it is designed as a feed-forward network at the test time. Their model predicts the distribution of possible colors for each pixel, and re-weighting the loss during the training prevents the discrimination of rare colors, returning more vivid results.

As in [42], the solution proposed by Larsson et al. [22] relies on a VGG network (training, in both cases, on the ImageNet dataset), but is coupled with hypercolumns (i.e., spatially localized multi-layer per-pixel descriptors). Given its hypercolumn descriptor, the method uses low-level and semantic representations, predicting the hue and chroma per-pixel histogram of the color distribution. The architecture is designed to link the color distribution with semantics by leveraging the features lying on several abstraction levels. Unlike the previously cited works, the architecture of Larsson et al. [22] is initialized, but not tied to the classification task, and fine-tuning is also possible on unlabeled data.

#### 2.2.2. Generative Adversarial Networks (GANs)

Just like CNN, the more recent generative adversarial networks (GANs) [49,50] use deep-learning-based generative modeling methods [51]. In these methods, algorithms learn the input data and their distribution in order to predict and generate new plausible examples fitting the original data’s distribution. In the adversarial process proposed by GANs and conditional GANs [52], generative models are estimated simultaneously, training two models: the Generator and the Discriminator models. The Generator apprehends the data distribution, while the Discriminator evaluates the sample’s probability of deriving from the training data (real) rather than from the Generator (fake). The generative process continues in an adversarial zero-sum game between the two models, progressively improving the prediction quality [53]. GANs usually employ CNNs for the Generator and Discriminator models. For the automatic colorization problem, during the generative process, additional inputs for the Generator are grayscale images rather than randomly generated noise, as in the general GANs formulation. The Discriminator is similarly conditioned from the additional grayscale images, along with the colored input from the Generator and the original data [54]. 

GANs-based colorization frameworks are progressively replacing more straightforward CNNs methods, despite their greater complexity [54,55,56,57,58,59,60,61,62]. These implementations adopt several learning strategies and network architectures, sometimes coupling the adversarial learning colorization with further perceptual or semantic information [59,60], or proposing a flexible framework for addressing several image-to-image translation problems [56]. 

As learning-based techniques can fail to predict colors when multiple objects are in the scene, the architecture of Su et al. [60] leverages an instance colorization network for extracting object-level features (using an off-shelf pre-trained object detector) and a similar network for the full-image features. Therefore, a fusion module is applied to predict the final colors. The colorization network introduced by Zhang et al. [42] is the backbone of this method.

On the other hand, Antic [57] introduced in “Deoldify” the NoGAN techniques for tackling Image to Image GAN training (a GAN training version optimized for reducing the training time). The designed architecture is based on the U-Net architecture, which is modified to introduce spectral normalization and self-attention into the model. Two models are available for image colorization, the Artistic and Stable models, while further implementation is dedicated to videos. The first uses a resnet34 backbone on U-Net, and the second uses a resnet101 on U-Net, respectively, emphasizing the layers’ depth and width on the decoder side. Although the Artistic version can generally return more detailed and vibrant colors, the less colorful Stable version was tested for its greater stability in predicting landscape and natural scenes.

### 2.3. Colorization of Aerial-Scale Images

Few works have focused on developing proper methods specifically designed for aerial scenarios [16,17,18], while further methods have been proposed for satellite imageries [63,64]. The solution proposed by Seo et al. [18] uses Random Forest Regressions and change detection to predict pixel color values. Change detection is performed between the input grayscale image and a colored reference image with similar seasonal features at the same location in a four-step workflow, where the last phase implies a color histogram adjustment. However, the method depends on the availability of the reference images of the same area with the same seasonal characteristics and the image’s orthorectification and registration quality. In Dias et al. [17], an adapted W-Net architecture is proposed for simultaneously segmenting images and predicting color values, stacking and bridging two U-Net architectures: the first encodes information on semantic classes, and the second decodes this information for predicting colors. Despite the promising results, the method has not been tested on original grayscale historical images, and its generalizability still has to be proven. The conditional GAN solution proposed by Poterek et al. [16] relies on a U-Net-like architecture for the Generator and a Patch GAN architecture for the Discriminator. However, all these methods designed for working with aerial-scale imageries are unavailable for tests and comparisons.

### 2.4. Benchmarking Methods

Some available state-of-the-art and recent methods (Section 2.2), based on both CNNs and GANs models, were selected and tested with aerial-scale imageries. In particular:

1. The joint learning of global and local image priors with the simultaneous classification approach proposed by Iizuka et al. [43];
2. The Larsson et al. [22] method, based on the exploitation of both low-level and semantic representations;
3. The colorization approach of Zhang et al. [42], addressed as a classification task;
4. The NoGAN technique, available in the Deoldify (Antic [57]) and relying on a modified version of U-NET;
5. The Instance-Aware colorization method of Su et al. [60], where the architecture leverages a network for extracting object-level and full-image features.

**Figure 2:**
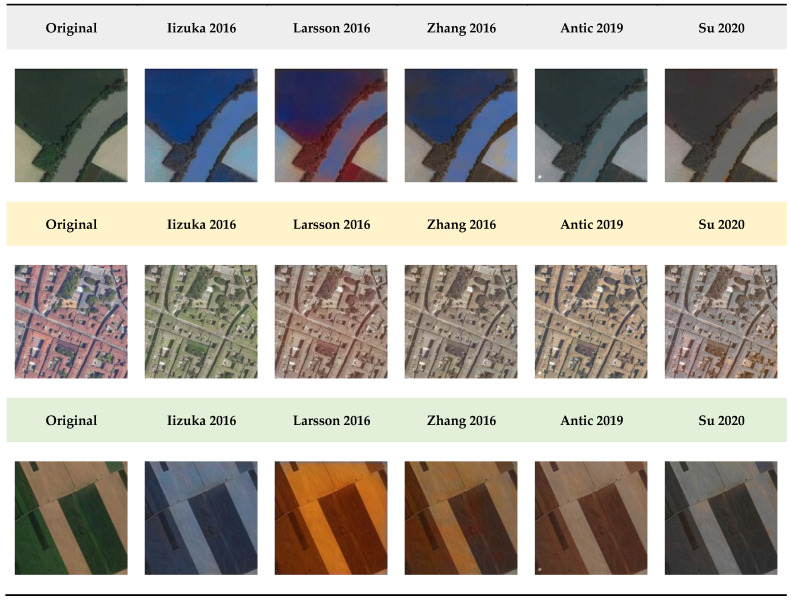
Some examples of colorization outputs obtained with the selected state-of-the-art techniques using aerial-scale images with urban and rural environments.

Actual RGB images were converted into grayscale, and the colorized results were used to verify their colorization capabilities. These methods failed, in most cases, at chrominance prediction for heterogeneous and rural environments captured from aerial platforms, as shown in Figure 2. Driven by these poor results, a new deep-learning architecture to handle the automatic colorization of historical aerial photos is presented in Section 3.

## 3. Proposed Method

A new colorization deep-learning approach, named “Hyper-U-NET” (Section 3.2), is hereafter presented. The method works in the L*a*b color space (Section 3.1) and was trained using a multi-scale training dataset composed of about 10,000 aerial image patches (Section 3.3). 

### 3.1. Color Space

The RGB space is the basic space with three components (red, green, and blue) diffusely employed in computer vision applications. However, for the automatic image colorization task, the YUV and CIELAB color spaces (the last introduced by the International Commission on Illumination—CIE—in 1976) are mostly preferred, covering the entire range of human color perception. As recently demonstrated by Ballester et al. [65], it cannot be concluded that one color space is always preferable in colorization applications, but the performance depends on the type of input images. For our Hyper-U-NET methodology, the L*a*b space, also used in the other methods tested in this work (Section 2.4), was selected for Hyper-U-NET, applying some modifications needed to handle the historical input images.

Also referred to as L*a*b, L indicates perceptual lightness, while the a * and b * axes range from green to red and from blue to yellow, respectively. The L, a*, and b* components are calculated by primarily converting RGB into the XYZ space. The L component, corresponding to the luminance percentage (from black to white), is derived by assigning a maximum weight to the green component and penalizing the blue one (Equation (1)):

L = Y = 0.2126 × R + 0.7152 × G + 0.0722 × B(1)

For the colorization of historical (scanned) aerial photographs, this formulation can be adjusted considering the signal transformation from analog to digital, following the BT.601 standard [66], where L is defined as follows (Equation (2)):

L = 0.299 × R + 0.587 × G + 0.114 × B(2)

Inspired by this formulation, we defined a new color space, the simplified L*a*b (sLab), starting from converting the RGB space into XYZ, as follows (Equations (3)–(5)):

X = 0.449 × R + 0.353 × G + 0.198 × B(3)

Y = 0.299 × R + 0.587 × G + 0.114 × B(4)

Z = 0.012 × R + 0.089 × G + 0.899 × B(5)

The L, a*, and b* components are finally calculated as follows (Equations (6)–(8)):

L = Y(6)

a* = (X − Y)/0.234(7)

b* = (Y − Z)/0.785(8)

### 3.2. Proposed Architecture

**Figure 3:**
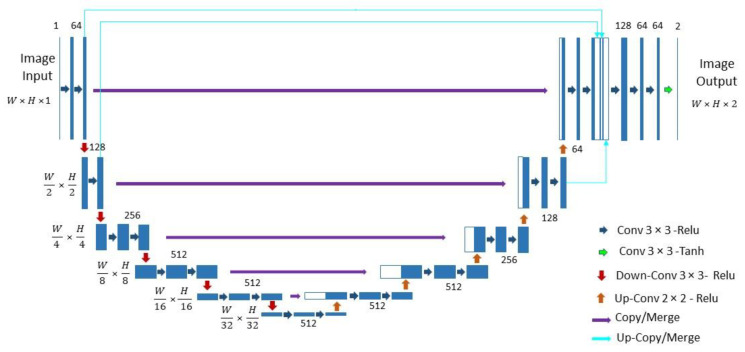
Architecture of the proposed Hyper-U-NET for the colorization of aerial grayscale images.

The developed solution for grayscale image colorization is named “Hyper-U-NET” (Figure 3). The architecture is a combination of a U-NET network [20] and the HyperConnections, inspired by the Hypercolumns technique [21,22].

#### 3.2.1. The U-NET Part

The U-NET architecture, originally implemented for fast and precise biomedical image segmentation, comprises two symmetric paths: an encoding/contracting path to capture context and a decoding/expanding path that enables precise localization. In the U-shaped architecture, high-resolution features from the contracting path are combined with the up-sampled outputs for handling the localization. Moreover, a large number of the feature channels also in the expanding path enables the propagation of context information to higher resolution layers. 

The architecture of the encoding part is a typical convolutional neural network (CNN), selected in our implementation to transfer the weights of the VGG16 network [1] to the contracting section. 

It is composed of six blocks, where each block is a group of 3 × 3 convolution layers (two or three layers) followed by a rectified linear unit (ReLu). A 2 × 2 max-pooling operation is applied at the end of each block (except the last one) to downsample the feature map by a factor of 2. 

The number of feature channels is fixed for the first block at 64, and doubles in the following blocks until 512, i.e., the maximum number of channels used in our network. 

The expanding/decoding part (right side) also includes six blocks (the first corresponds to the last of the encoding part). Each block comprises three 3 × 3 convolution layers followed by a rectified linear unit (ReLu) and ends with a 2 × 2 upsampling operation (except the last one). The number of feature channels is maintained at 512 for the first three blocks and is then halved until reaching 64.

Unlike the fully convolutional approach, the final feature maps of each block of the encoding part (just before the max-pooling layer) are concatenated with their corresponding feature maps from the contracting path (see Figure 3). This “skip connection” step is a helpful feature of the U-NET architecture, used to solve the known degradation problem and to ensure future reusability.

#### 3.2.2. The HyperConnections Part

Our U-NET-like architecture is further expanded by means of HyperConnections, inspired by the hypercolumns [21] introduced for object segmentation and fine-grained localization tasks. Hypercolumns are per-pixel descriptors, i.e., vectors of activation of all CNN layers located above the pixels. This technique allows for precisely exploiting spatially localized information contained in different CNNs units. In our implementation, HyperConnections are defined at the 2D feature maps level. They are up-sampled to the final layer size and concatenated with the last feature maps of the expanding path. At the end of the network, three “3 × 3 convolutions and ReLu” were added with decreasing the number of channels. The figure shows an example of the network architecture merging three HyperConnections (heavenly arrows) with the last feature map of the expanding path, with two of them up-sampled to the final layer size. This number can be increased or decreased, taking into account the number of training images, the complexity of the confronted problem, and the GPU/memory capacity. Figure 3 shows the optimal configuration regarding the quality of results and the computational efficiency found in our experiments.

### 3.3. Training Data

About 10,000 aerial image patches were collected and used for training our Hyper-U-Net network (Section 3.2). 

**Figure 4:**
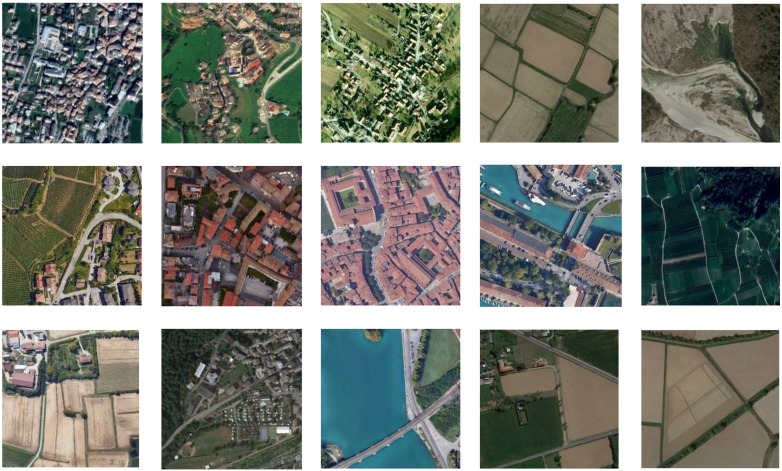
Some examples from the multi-scale training dataset collected and shared for the colorization of historical aerial images: the patches feature different radiometric properties and depict several built and natural environments.

Data can be downloaded from the link inserted on the GitHub page (https://github.com/3DOM-FBK/Hyper_U_Net) [accessed on 27 September 2022]. The patches (512 × 512 pixels) depict urban, rural, and natural scenarios (Figure 4), captured at different scales, and are heterogeneous in terms of their radiometric properties. To achieve plausible results with the colorization of historical aerial photos, varied built and natural environments were considered: different seasons and shadow conditions, several tones for vegetated areas, various roof types and colors (generally omitting industrial areas), water areas, etc. 

For training the Hyper-U-NET (Section 3.2), some image data augmentation (flipping, rotation, and contrast/brightness modifications) was also applied to help the learning process to improve the prediction results and increase the network robustness. The complete evaluation with metrics (Section 4) was done on some 50 actual images (converted in grayscale and re-colorized), as some state-of-the-art methods perform the colorization using one image at a time (manually uploaded to an online processing system).

## 4. Experiments and Results

### 4.1. Evaluation Metrics

Color difference evaluation is a complex and investigated task [67,68,69,70]. Studies in this field aim to identify a comprehensive formulation for objectively quantizing color differences, considering the influence of many factors on color perception and comparison. Therefore, some mathematic models have been developed to reproduce the color perception experience, mainly designed in three-dimensional spaces (as the three types of receptors in the human eyes). 

Following the literature, the metrics adopted in this work for handling this complex evaluation task are as follows:

(1) The ∆*E*2000 (DeltaE-CIEDE2000) (Equation (9)):

(9) $$\Delta E_{00}=\sqrt{{\left(\frac{\Delta L^{\prime }}{k_{L}S_{L}}\right)}^{2}+{\left(\frac{\Delta C^{\prime }}{k_{C}S_{C}}\right)}^{2}+{\left(\frac{\Delta H^{\prime }}{k_{H}S_{H}}\right)}^{2}+R_{T}\frac{\Delta C^{\prime }}{k_{C}S_{C}}\frac{\Delta H^{\prime }}{k_{H}S_{H}}}$$

This is an expanded and updated version of previous mathematic formulations for determining the color difference, where *L* is weighted depending on the brightness of the color value range [71]. The smaller ∆*E*2000, the lower the difference between the reference and target colors.

(2) The mean absolute error (*MAE*) (Equation (10)), i.e., the average of the absolute differences between the observed and predicted color values, defined as follows:

(10) $$MAE=\frac{1}{N}\overset{N}{\underset{i=1}{\sum }}|\;y-\hat{y}|$$

Small *MAE* values indicate a major color similarity.

(3) The peak signal-to-noise ratio (*PSNR*) [72] (Equation (11)), defined as:

(11) $$PSNR=10\;\log\left(\frac{3mn{\left(MAX\right)}^{2}}{\sum_{RGB}\sum_{i=0}^{m-1}\sum_{j=0}^{n-1}\lfloor u\left(i,j\right)-u_{0}{\left(i,j\right)\rfloor }^{2}}\right)$$

where MAX is the maximum possible pixel value (255) and $\underset{RGB}{\sum }()$ is the summation over the red, green, and blue bands. Higher *PSNR* values indicate a higher quality of the predicted image.

(4) The Structural Similarity Index Measure (*SSIM*) [73] (Equation (12)), defined as:

(12) $$SSIM\left(x,y\right)=\frac{\left(2{µ}_{x}{µ}_{y}+c_{1}\right)\left(2s_{xy}+c_{2}\right)}{\left({µ}_{x}^{2}+{µ}_{y}^{2}+c_{1}\right)\left(s_{x}^{2}+s_{y}^{2}+c_{2}\right)}$$

*SSIM* values closer to 1 indicate a higher image similarity.

### 4.2. Ablation Experiment

In the ablation study hereafter presented, the contribution of the newly introduced HyperConnections part (Section 3.2.2) to our network is primarily investigated.

Ablation experiments were conducted considering the following:

(a) U-NET: a standard U-NET model trained on our dataset. The model has the same configuration as our Hyper-U-NET, except for the HyperConnections and the last extra three layers;
(b) Hyper-U-NET1: the model proposed in the paper, trained from the beginning on our dataset;
(c) Hyper-U-NET2: unlike the previous case, it is finetuned based on the best model found on the U-NET part.

**Figure 5:**
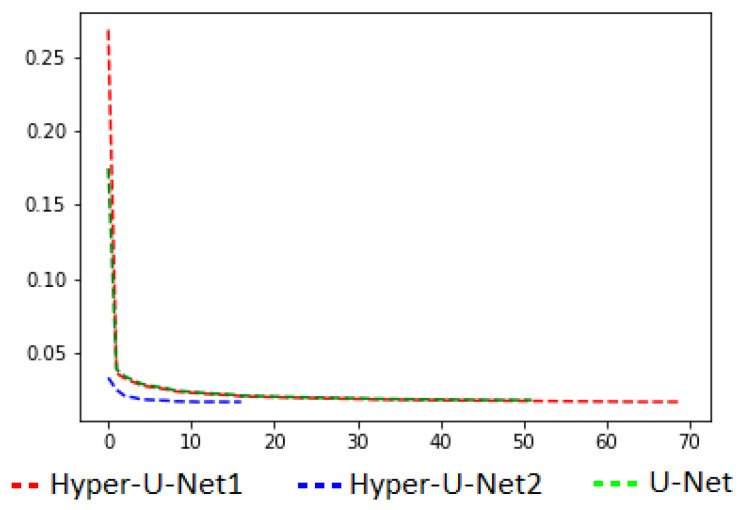
Loss function curve comparisons.

For training, the initial learning rate equaled 10^−4^, and it decreased until the minimum values of 10^−7^ were fixed. The mean absolute error (*MAE*) was adopted as a loss function (Figure 5), while the ADAM method [74] was adopted for optimizing the model. The maximum number of epochs was set to 200, and the training stopped when no evolution was evident on the loss values. The GPU used was an NVIDIA Tesla V100S PCIe 32GB. 

A quantitative evaluation of the three different models is presented in Table 1, as testing images fifty actual aerial images converted into grayscale and then re-colorized as the testing images. The results show a slight improvement in the metrics for both Hyper-U-NET implementations compared with the standard U-NET model. 

A comparison of the training and prediction times is offered in Table 2. While the training time was calculated with 10,000 image patches (512 × 512 pixels), the prediction time was the time the model spent to predict an image of 512 × 512 pixels. The results show that the U-NET model had the best performance for both the training and the prediction times. 

Furthermore, it required only 47 epochs to converge to the optimum solution (the best model), with an average of 19.5 min for each epoch, while Hyper-U-NET1 required 65 epochs with an average time of 28 min for each epoch. The Hyper-U-NET2 model, trained using U-NET weights as initial values, required only 12 epochs, 28 min per epoch, and 20.7 h for training. This time was the sum of the training time of the U-NET (15.1 h) and the Hyper-U-NET2 (5.6 h).

**Figure 6:**
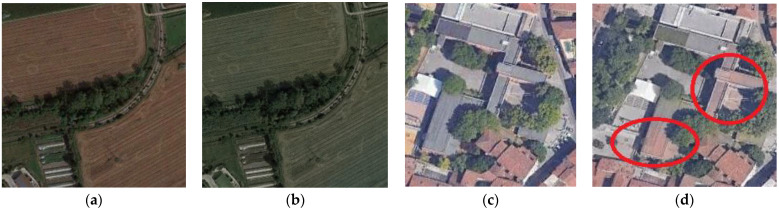
Reference actual aerial images converted in grayscale and used in our colorization tests (**a**,**c**) and examples of incorrect prediction (**b**) and ambiguities (**d**) using the U-NET model.

Although the metrics (Table 1) showed slight improvements with our implementations, and the U-NET model was favored regarding the training and prediction times (Table 2), visual colorization outputs with this model proved its ineffectiveness and several ambiguities with the tested aerial images (Figure 6). These results confirm the benefits of using the HyperConnections for feature preservation during the U-NET training and of the last extra layers to improve the quality of the results. Hyper-U-NET2 (referred to in the article as Hyper-U-NET) was the model finally selected in this contribution.

### 4.3. Colorization of Historical Aerial Images

**Figure 7:**
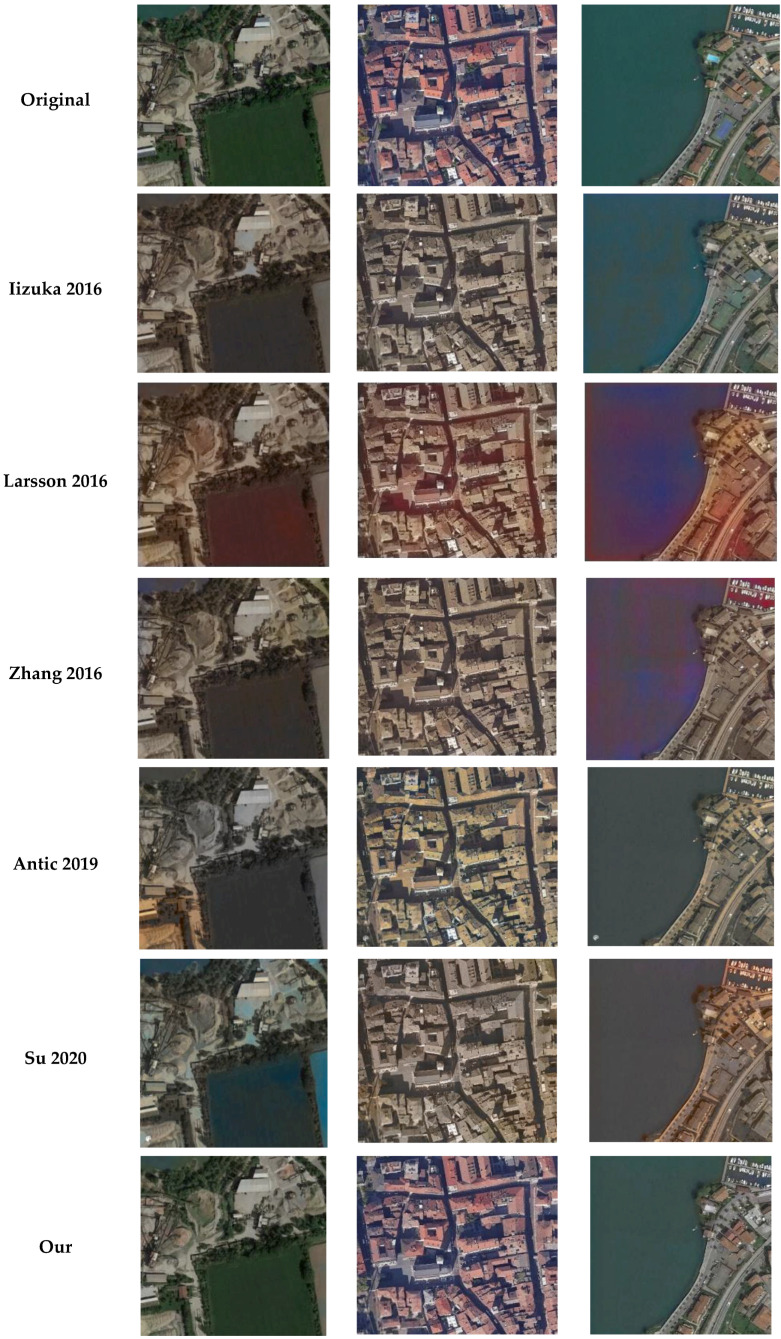
Some colorization outputs, comparing the proposed Hyper-U-Net method with state-of-the-art methods on actual aerial images converted into grayscale.

A visual and metric assessment of some colorization outputs is hereafter presented, testing the CNN and GAN algorithms presented in Section 2.4 and the proposed Hyper-U-NET network (Section 3.2). For the evaluation, considering the unavailability of ground truth data for historical photographs, some 50 actual aerial images were converted into grayscale and were re-colorized. Some colorization results for urban and rural areas are shown in Figure 7, whereas the metrics are reported in Table 3. 

The implemented Hyper-U-Net outperformed the existing and available colorization methods in almost all of the considered metrics. 

Visual comparisons (Figure 7) confirmed the capability of the implemented procedure to generate acceptable results and to correctly predict colors in the aerial scenarios. 

**Figure 8:**
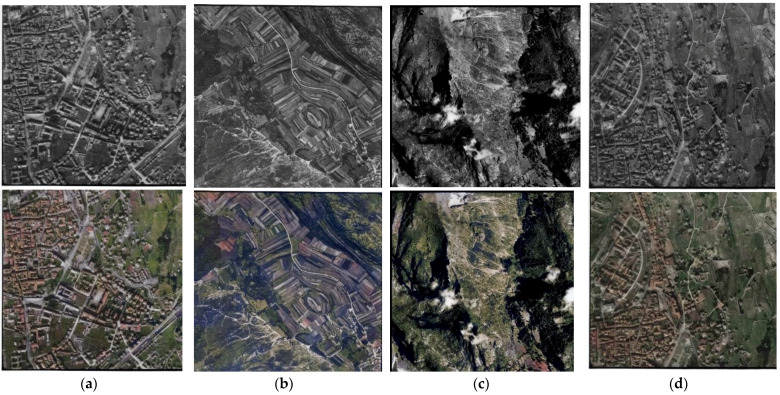
Some examples of historical grayscale images (first row) available in the TIME benchmark [19] colorized with the proposed Hyper-U-NET (second row), depicting mostly urban (**a**,**d**), rural (**b**), and mountainous (**c**) environments.

Some further visual results obtained on the historical aerial images belonging to the TIME benchmark [13] (https://time.fbk.eu [accessed on 27 September 2022]), acquired in Italy between 1944 and 1945, and colorized with the proposed Hyper-U-Net, are shown in Figure 8.

## 5. Discussion

Automatic color prediction is a very complex image processing task, just like the proper evaluation of colorization outputs. Especially when the learning models exploit semantics, correct object recognition and representation are crucial for producing an adequate chromatic transformation. In every case, some ambiguities are created when multiple colorization options are possible for the same object (e.g., red or gray roofs, a wide range of shades of green or brown distinguishing several agricultural destinations). This problem is mainly present in GANs methods, where mode collapse and failures can occur when the prediction of classes and semantics has multiple possibilities.

Regarding prediction and color difference evaluation, the need and the complexity of objectively describing and measuring some properties related to the perceptive sphere have driven many investigations and mathematic formulations for conducting this assessment. The available metrics, however, can sometimes deliver inconsistent results compared with what is perceived, as also noted by other authors [16]. Frequently, more unsaturated outputs seem to be preferred by these metrics.

The tested state-of-the-art methods were proven to hardly adapt to bigger-scale images, being designed and trained for working primarily in terrestrial contexts. At the same time, retraining these networks with our images was excluded, considering the difficulty of identifying consistent settings for all parameters among the methods and, in some cases, the absence of open-source code.

In order to supply the unavailability of other methods for the colorization of historical aerial images, Section 3 presented a newly developed architecture devoted to this scope. Hyper-U-NET combines diverse existing techniques and approaches, and several network configurations can be implemented (through the hypercolumns combination) considering specific GPU capacities and colorization problems.

The method delivered outstanding results with actual images converted in grayscale and re-colorized (Figure 7 and Table 3), being able, in most cases, to correctly predict key image feature colors, such as roofs, rivers, sea, and vegetation.

**Figure 9:**
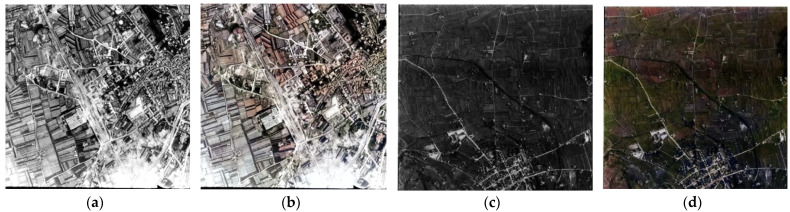
Colorization outputs (**b**,**d**) Hyper-U-NET with historical photos affected by unbalanced contrast/brightness levels (**a**,**c**), and depicting an urban area (**a**) and the surrounding countryside and a mostly rural (**c**) environment.

On the historical aerial image sets (Figure 8), still plausible results were achieved in many cases, although the lack of ground truth data made the evaluation in this case more complex and only qualitative. The quality of colorization outputs with analog aerial imageries that resulted strongly conditioned and affected by the quality of the input images, mainly defined by the quality of the capturing cameras and acquisition settings, as well as the scanning process. Hyper-U-NET was tested on heterogeneous images in terms of resolution, exposure, contrast, and brightness levels. When images presented a poor or unbalanced distribution of these components, the network returned poor colorization results, demonstrating the method’s limitations and quality dependency (Figure 9).

Extremely bright or dark regions often generate ambiguous or incorrect colorization results, because the brightness range changes with the terrain, the flying height, and the spectral features of the captured objects. However, when archival digital images featured correct exposure and balanced contrast/brightness levels, Hyper-U-NET provided a good chrominance distribution and a wide range of colors for the elements captured in the scenes (such as roofs, vegetated and cultivated lands, streets, and snowy and mountain areas). 

## 6. Conclusions and Future Works

The article explored and examined deep-learning techniques for handling the automatic colorization of grayscale aerial images. Color prediction outputs of some existing CNN and GAN implementations were evaluated with aerial-scale pictures, and a new architecture was proposed for handling the colorization of historical aerial photographs. 

The proposed Hyper-U-NET method returned satisfactory colorization outputs in many scenarios, from a qualitative and quantitative point of view, although some failures occurred in the case of low image quality. 

Further tests are planned to analyze achievable improvements by applying image enhancement and image-restoration techniques before applying the colorization methodology. Other investigations will deepen the effectiveness and the benefits of employing archival colorized compared with grayscale images for handling further processing tasks (e.g., object recognition and classification) and multi-temporal analyses. 

Finally: the comparison of several colorization outputs of Hyper-U-NET with historical data and working with different color spaces could also drive and help improve further implementation of the method.

## Figures and Tables

**Table 1:** Results of the ablation experiments with three different models. The best results of each column are in bold.

	∆*E* 2000 ↓	*MAE* ↓	*PSNR* ↑	*SSIM* ↑
U-NET	0.797	4.315	32.9	98.32
Hyper-U-NET1	0.735	4.058	33.302	98.46
Hyper-U-NET2	**0.723**	**3.957**	**33.508**	**98.47**

**Table 2:** Training and prediction time consumption of the different models.

	Training Time (h)	Prediction Time (s)	Epochs
U-NET	15.1	0.132	47
Hyper-U-NET1	30.3	0.149	65
Hyper-U-NET2	20.7	0.149	12

**Table 3:** Average metric values for some 50 aerial images colorized with some existing deep-learning methods and the proposed Hyper-U-Net method.

	∆*E* 2000 ↓	*MAE* ↓	*PSNR* ↑	*SSIM* ↑
**Iizuka et al. [43]**	1.683	10.506	26.257	0.955
**Larsson et al. [22]**	1.777	34.309	21.273	0.913
**Zhang et al. [42]**	1.620	11.721	25.318	0.951
**Antic [57]**	1.716	10.257	25.749	0.946
**Su et al. [60]**	1.604	10.413	26.200	0.949
**Our**	**0.764**	**3.987**	**33.287**	**0.980**

## Data Availability

The code for the colorization and the collected training datasets are available on our GitHub page (https://github.com/3DOM-FBK/Hyper_U_Net) [accessed on 27 September 2022].
